# A 3D Point Cloud Classification Method Based on Adaptive Graph Convolution and Global Attention

**DOI:** 10.3390/s24020617

**Published:** 2024-01-18

**Authors:** Yaowei Yue, Xiaonan Li, Yun Peng

**Affiliations:** 1 School of Computer and Information Engineering, JiangXi Normal Universtity, Nanchang 330224, China; pengyun@jxnu.edu.cn; 2 School of Information Engineering, East China University of Technology, Nanchang 330013, China; 13776860791@163.com

**Keywords:** global attention, adaptive graph convolution, adaptive kernels, point cloud classification

## Abstract

In recent years, there has been significant growth in the ubiquity and popularity of three-dimensional (3D) point clouds, with an increasing focus on the classification of 3D point clouds. To extract richer features from point clouds, many researchers have turned their attention to various point set regions and channels within irregular point clouds. However, this approach has limited capability in attending to crucial regions of interest in 3D point clouds and may overlook valuable information from neighboring features during feature aggregation. Therefore, this paper proposes a novel 3D point cloud classification method based on global attention and adaptive graph convolution (Att-AdaptNet). The method consists of two main branches: the first branch computes attention masks for each point, while the second branch employs adaptive graph convolution to extract global features from the point set. It dynamically learns features based on point interactions, generating adaptive kernels to effectively and precisely capture diverse relationships among points from different semantic parts. Experimental results demonstrate that the proposed model achieves 93.8% in overall accuracy and 90.8% in average accuracy on the ModeNet40 dataset.

## 1. Introduction

With the continuous advancement of various sensors and image matching technologies, three-dimensional (3D) point clouds have found widespread applications in various domains. Effective classification of point clouds plays a crucial role in fields such as autonomous driving, robot navigation, augmented reality, and 3D reconstruction. However, due to the irregularity and sparsity inherent in 3D point clouds, classifying them in complex environments is by no means a straightforward task. Furthermore, the density of point clouds can vary depending on the sampling interval and range of the laser scanner, while severe occlusions between objects during the scanning process can result in incomplete coverage of object surfaces. These challenges pose significant hurdles in the classification of 3D point clouds.

As previously mentioned, applying standard convolutional neural networks directly to three-dimensional point clouds is infeasible due to their unordered and unstructured nature. Some researchers have started to regularize point clouds to draw insights from the experience of two-dimensional semantic segmentation networks. In the literature [1], the authors presented the groundbreaking work PointNet [1], which operates directly on irregular point clouds, utilizes shared Multi-layer Perceptrons (MLPs) to learn point features, and employs symmetric pooling functions to capture global features. Building upon PointNet [1], subsequent scholars have proposed a series of point-wise MLP methods such as PointNet++ [2], Frustum-PointNet [3], PCNN [4], DGCNN [5], and PointWeb [6]. However, the use of shared MLPs for extracting 3D point cloud features may not adequately capture local geometric characteristics within the point cloud and overlooks interactions between points. Zhang [7] introduced an interpretable point cloud classification learning method, PointHop, which primarily employs spatial partitioning to address the data challenges in unordered point clouds and explores ensemble methods to enhance classification performance. Ben-Shabat [8] introduced an intuitive three-dimensional point cloud representation called Fisher Vectors (3DmFV) using grids to design novel network architectures for real-time point cloud classification. 3DpointCapsNet [9] proposed a 3D point capsule network that preserves the spatial arrangement of input data and designs a 2D latent space, bringing improvements to several common point cloud-related tasks.

Nonetheless, the conventional Multilayer Perceptron (MLP) approach is subject to inherent limitations when addressing global feature interactions between points, owing to the mutual independence of neurons. Moreover, MLP exhibits suboptimal modeling efficacy in the context of long-range dependency relationships. The pioneering Transformer model, introduced by Vaswani [10], initially garnered remarkable success in the domain of Natural Language Processing (NLP). Subsequently, Wang [11] introduced the innovative Point-Transformer, effectively managing variable length data and global information, resulting in enhanced classification accuracy and generalization capabilities.Notably, it achieved a notable stride in modeling point-to-point interaction. He [12] engineered the PointCloudTransformer, harnessing Transformer’s self-attention mechanisms to capture the global information of point cloud data, while employing Convolutional Neural Networks for handling local information, thus achieving highly efficient classification. However, Transformers prove less effective in capturing the topological structural characteristics of point clouds.

**Figure 1:**
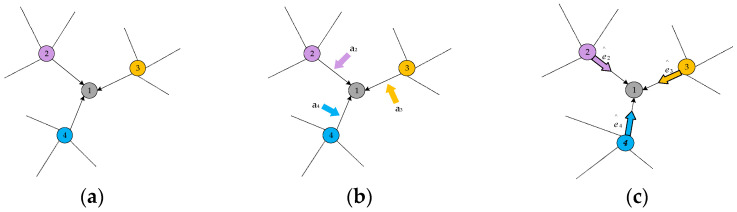
Diagram of adaptive kernel and fixed kernel in convolution. (**a**) Graph Convolution; (**b**) Graph Convolution with Weights; (**c**) Adaptive Kernel.

To enable each point to capture a broader context and obtain richer local hierarchies, some scholars have proposed utilizing graph structures for point cloud analysis. GraphCNN [5,13,14,15,16] represents point clouds as graph data based on spatial/feature similarities between points and extends 2D convolution on images to 3D data. To handle unordered point sets with varying neighborhood sizes, standard graph convolution employs shared weight functions for each pair of points to extract corresponding edge features. This results in a fixed/isotropic convolution kernel that is applied to all pairs of points, overlooking their distinct feature correspondences. Intuitively, for points from different semantic parts of a 3D point cloud (such as adjacent points in Figure 1), the convolution kernel should be able to differentiate them and determine their varying contributions. To address this limitation, several dedicated networks have been introduced, including a neighborhood feature pooling-based approach [2], attention-based aggregation [17], and local global feature fusion methods [5,18,19]. By assigning appropriate attention weights to neighboring points, these approaches attempt to identify their varying importance during convolution. However, these methods still fundamentally rely on fixed kernel convolutions since attention weights are applied to similar features obtained (as indicated by the black arrows in Figure 1b). As illustrated in Figure 1a, standard graph convolution applies a fixed and isotropic kernel (black arrows) to compute features for each point. Part b Based on these features, several attention weights are assigned to determine their importance. In contrast to the previous two, ‘ c ’, generates an adaptive kernel ‘ $\hat{e_{i}}$ ’, unique to learning features for each point.

**Figure 2:**
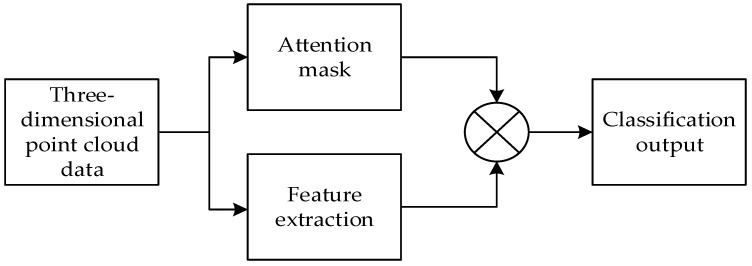
Att-AdaptNet model.

To address this, we propose a novel deep learning model called Att-AdaptNet (Figure 2). In this paper, featuring attention-based global feature masking and channel weighting, corresponding to the global attention module and adaptive graph convolution (see Figure 2). The entire end-to-end model takes 768 to 1408 point clouds as input for classification learning. There are two primary branches in this model. The first branch focuses on the influence of each local point, thus producing a global mask at the branch’s end that weights the contribution of each point to the point cloud features. To capture fine-grained regions on the point cloud, the global features are multiplied by the mask to obtain the final attention-based features. The other branch employs adaptive graph convolution to generate adaptive kernels, replacing the aforementioned isotropic kernels (see Figure 1c). The adaptive kernels achieve adaptivity during convolution operations, as opposed to merely assigning different weights to adjacent points.

The experiments demonstrate that, on the widely used ModelNet40 benchmark dataset, Att-AdaptNet outperforms many existing models. To ensure a fair comparison, following the practice of most deep learning papers, the proposed approach is benchmarked against other models on ModelNet40. The key reason for the superiority of the Att-AdaptNet lies in its innovative introduction of attention mechanisms into point cloud feature extraction, where each point plays a unique role in describing the overall structure. Thus, the model assigns individual weights to each point during the feature integration stage, while also emphasizing crucial feature channels representing intrinsic geometric information in high-dimensional space. The main contributions of this chapter are summarized as follows:

(1) We propose a novel 3D point cloud classification method, named Att-AdaptNet, based on attention and adaptive graph convolution. This method can directly process raw point clouds and employs attention mechanisms through global feature masking and adaptive graph convolution to focus on feature regions.
(2) We utilize adaptive graph convolution to extract global features from 3D point clouds, effectively and precisely capturing diverse relationships among points from different semantic parts.
(3) The Att-AdaptNet is trained and tested on the ModelNet40 benchmark dataset, achieving a classification accuracy of 93.3%. It demonstrates significant improvements in performance compared to other methods.

## 2. Related Works

Self-attention networks have garnered significant attention for their ability to extract discriminative features of interest, allowing models to identify the focal points. Thus far, self-attention-based models have found wide applications in tasks such as machine translation, caption generation [20], speech recognition [21], and adversarial networks [22], among others. The self-attention mechanism is designed to enable the network to learn context beyond the receptive field. One of the initial successful incorporations of this mechanism into CNNs was witnessed in the Squeeze-and-Excitation network [23].

Petar Veličković introduced the Graph Attention Mechanism and constructed the corresponding Graph Attention Network (GAT) [24]. It primarily utilizes self-attention to obtain attention coefficients, normalizes them, and then linearly combines them with the corresponding feature vectors, resulting in the final output features. PCAN [17] proposed an attention mechanism for local feature aggregation to distinguish positively contributing local features. However, this method mainly employs a point-wise structure to extract local features, which does not particularly focus on local geometric structures. GAC [16] introduced an attention mechanism based on the PointNet architecture, where attention weights learned from neighboring points can capture discriminative features, and this method achieved good performance. Chen et al. [25] presented the GAPNet model, which aggregates attention features for each point in the neighborhood using a multi-head attention mechanism and applies stacked MLP layers to capture local geometric features from the original point cloud, achieving promising results. Yang et al. [26] developed the Point-Attention Transformer (PAT) to model interactions between points, employing parameter-efficient Group Shuffle Attention (GSA) instead of expensive multi-head attention mechanisms.

Influenced by attention mechanisms and pyramid pooling, several methods have been proposed to better capture local geometric information, GGM-Net [27] introduced a Graph Geometry Moment Convolutional Neural Network that learns local geometric features from the geometric moment representations of local point sets to better capture local geometric information. AGCN [28] avoids the use of shared spectral kernels and instead assigns a customized Laplacian graph to each sample, providing an objective description of its graph convolution topology. Li [29] aimed to extract precise pixel-level attention from high-level features obtained from CNNs. They proposed the Feature Pyramid Attention (FPA) module, which effectively increases the receptive field and aids in the classification of small objects by embedding context features of different scales in a pixel prediction framework based on FCN. PyramNet [30] primarily designed two new operators, the Graph Embedding Module (GEM) and the Pyramid Attention Network (PAN). GEM projects point clouds onto graphs and utilizes covariance matrices to explore relationships between points, enhancing the model’s ability to represent local features. PAN assigns strong semantic features to each point, preserving fine-grained geometric features as much as possible. Wang et al. [16] introduced GACNN, an end-to-end encoder-decoder network that captures multi-scale features of point clouds, achieving more accurate point cloud classification. 

## 3. Model Construction

In recent years, deep neural networks have emerged as a primary tool for image analysis. Deep learning, due to its capacity for large-scale learning, has also gained popularity in the realm of 3D point cloud classification. Since the introduction of PointNet [1], recent works have focused on extracting global features of point sets by grouping and aggregating features of all individual points. However, these approaches are limited to detecting structural differences between different objects. Therefore, this paper proposes a novel deep learning model called Att-AdaptNet.

### 3.1. Adaptive Graph Convolution Module

**Figure 3:**
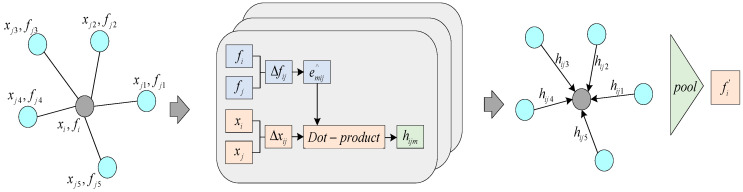
The operation process of the AdaptConv module.

The adaptive graph convolution is an extension of graph convolution, and the configuration of the adaptive convolution module in this paper is the same as that in AdaptConvNet [28]. The structure of this module is illustrated in Figure 3. Let $X=\left\{x_{i}|i=1,2,\ldots ,N\right\}\in {\mathbb{R}}^{N\times 3}$ be the input point cloud, with corresponding features defined as $F=\left\{f_{i}|i=1,2,\ldots ,N\right\}\in {\mathbb{R}}^{N\times D}$ . Here, $x_{i}$ represents the ( x , y , z ) coordinates of the i -th point, and in general, it can be augmented with vectors of other attributes such as normals and colors. Then, a graph is constructed for each point, including self-loops, by considering the k-nearest neighbors (KNN) for each point, resulting in a directed graph G(V, E) where $V=\left\{1,2,\ldots ,N\right\}$ and E⊆V×V represents a set of edges. Given the input feature dimensions, the AdaptConv [24] layer aims to generate a new set of M-dimensional features with the same number of points while attempting to more accurately reflect local geometric features than previous graph convolutions.

The adaptive kernel, denoted as ${\hat{e}}_{ijm}$ , is generated from the input features $\Delta f_{ij}$ of a pair of points on the edge. It is then convolved with the corresponding spatial input $\Delta x_{ij}$ to produce the corresponding edge feature $h_{ijm}$ . All dimensions of $h_{ijm}$ are concatenated to produce the edge feature $h_{ij}$ , which is finally pooled to output the feature $f_{i}^{\prime }$ of the central point. What sets AdaptConv apart from other graph convolutions is that the convolution kernel for each pair of points is unique. Here, $x_{i}$ represents the central point in the graph convolution, and $N\left(i\right)=\left\{j:\left(i,\;j\right)\in E\right\}$ is a set of points in its neighborhood due to the irregularity of point clouds, previous methods often used a fixed kernel function for $x_{i}$ ’s neighbors to capture the geometric information of the patch. However, different neighborhoods reflect different features of $x_{i}$ , especially when $x_{i}$ is located in prominent regions such as corners or edges. A fixed kernel may lead to geometric representations generated by graph convolution that are not well-suited for classification.

Therefore, this chapter aims to capture unique relationships between each pair of points using an adaptive kernel. For each channel in the output M-dimensional features, AdaptConv dynamically generates a kernel based on the point features $\left(f_{i},\;f_{j}\right)$ , as follows Equation (1):

(1) $$\hat{e_{ijm}}=g_{m}(\Delta f_{ij}),j\in \mathbb{N}(i)$$

Here, m=1, 2,…,M represents one of the M output dimensions corresponding to a single filter defined in AdaptConv. To combine the global shape structure captured in the local neighborhood [6] with feature differences, this chapter defines $\Delta f_{ij}=\left[f_{i},\;f_{j}-f_{i}\right]$ as the input feature for the adaptive kernel, where [·, ·] denotes concatenation operation $g\left(\cdot \right)$ is a feature mapping function, and in this case, a multi-layer perceptron is used.

Similar to the computation of 2D convolution, convolution is performed by taking D input channels and their respective filter weights to obtain one of the M output dimensions. Then, convolution is applied between the adaptive kernel and the corresponding points $\left(x_{i}\;,x_{j}\right)$ , as shown in Equation (2):

(2) $$h_{ijm}=\sigma <\hat{e_{ijm}},\Delta x_{ij}>$$

In Equation (2), $\Delta x_{ij}$ is defined as $\left[x_{i},\;x_{j}-x_{i}\right]$ , <·, ·> denotes the inner product of two vectors, and $h_{ijm}\in \mathbb{R}$ is subject to a non-linear activation function σ. As shown in Figure 3, the m-th adaptive kernel ${\hat{e}}_{ijm}$ combines with the spatial relation $\Delta x_{ij}$ of the corresponding point $x_{j}\in {\mathbb{R}}^{3}$ . The size of the kernel should match in the dot product, meaning the feature mapping $g_{m}:{\mathbb{R}}^{2D}\to {\mathbb{R}}^{6}$ , as mentioned earlier. This allows spatial positions in the input space to be effectively incorporated into each layer and combined with features extracted dynamically from the kernel. The $h_{ijm}$ from each channel is summed together, generating edge features $h_{ij}=\left[h_{ij1},\;h_{ij2},\ldots ,h_{ijm}\right]\in R^{M}$ between points $\left(x_{i},\;x_{j}\right)$ . Finally, the output feature of the central point is defined by applying an aggregation function to all edge features in the neighborhood:

(3) $$h_{ijm}=\sigma <\hat{e_{ijm}},\Delta x_{ij}>$$

In Equation (3), max represents a channel-wise maximum pooling function. To summarize, the convolutional weights for AdaptConv are defined by Equation (4):

(4) $$\Theta =(g_{1},g_{2},\ldots ,g_{M})$$

In this experiment, AdaptConv generates an adaptive kernel for each pair of points based on their respective features $\left(f_{i},f_{j}\right)$ . Then, this kernel, denoted as ${\hat{e}}_{ijm}$ , is applied to point pairs $\left(x_{i},x_{j}\right)$ to describe their spatial relationship in the input space. In other cases, the input can be $x_{i}\in {\mathbb{R}}^{E}$ , which includes additional dimensions representing other valuable point attributes, such as point normals and colors. By modifying the adaptive kernel to $g_{m}:{\mathbb{R}}^{2D}\to {\mathbb{R}}^{2E}$ , AdapConv can capture relationships between feature dimensions and spatial coordinates from different domains. In this chapter’s experiments, spatial positions are used as the default input in the convolution. Instead of using $\Delta x_{ij}$ , ∆ $f_{ij}$ is employed, and a pair of points’ adaptive kernels are designed to establish relationships between their current features $\left(f_{i},\;f_{j}\right)$ at each layer. This allows the kernel to adapt to the features from the previous layer, extracting feature relationships. It is a more direct solution, similar to other convolutional operators, as it generates a new set of learned features from the features of the previous layer in the network.

After two layers of AdaptConv and two layers of graph convolution, specifically following the output of the final layer, the model further utilizes a shared MLP (MLP $h_{\Theta }^{g}$ ) and an SE-1d block to obtain global feature representation g . The computation process is illustrated in Equation (5):

(5) $$g=F_{SE}(h_{\Theta }^{g}f_{i}^{\prime })\in R^{N\times C^{out}}$$

### 3.2. Global Attention

For each $x_{i}$ , a subset is defined with $x_{i}$ as the center, and k − 1 of the closest points excluding the center $x_{c}$ are selected. Thus, the KNN query for $x_{c}$ can be calculated as shown in Equation (6):

(6) $$F(x_{c})=\{x_{j}|∥x_{j}-x_{c}{∥}_{2}\leq ∥x_{c}-x_{ij}∥\}\in R^{k\times c}$$

where $x_{k}$ represents the k-th closest point to $x_{c}$ , calculated using a kNN query. Thus, the grouped input can be represented as shown in Equation (7).

(7) $$\{F(x_{i})|x_{i}\in x\}\in R^{N\times K\times C}$$

**Figure 4:**
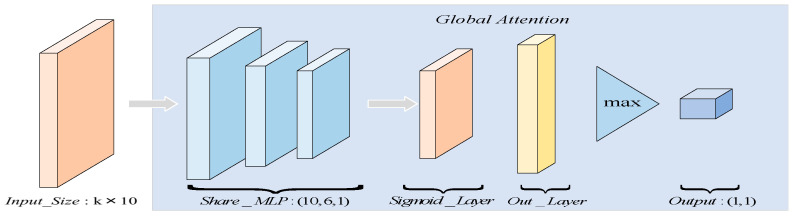
Global attention module.

The input to this module differs from the AdaptConv module. The Global Attention Module has additional geometric features, and this additional output is represented in the following form as shown in Equation (8):

(8) $$x_{i}^{input}=\{x_{i},x_{j},x_{j}-x_{i},∥x_{j}-x_{i}{∥}_{2}\}\in R^{k\times 10}$$

where $x_{i}\in x$ , $\left|\left|\cdot \right|\right|$ denotes the Euclidean distance, and k represents a set of points’ count. The structure of the Global Attention Module is depicted in Figure 4.

In this module, similar to channel attention in SENet [31], two 1 × 1-sized 2D convolutional layers are used to reduce the dimensionality of the grouped features (the input to this module), and a sigmoid function is employed to generate a soft attention mask. For a specific point cluster $F\left(x_{i}\right)$ centered at $x_{i}$ , the calculation of the importance of $x_{i}$ is defined by Equation (9):

(9) $$x_{i}^{GA}=\underset{j\in [1,k]}{\max}Sigmoid(h_{\theta }(x_{i}^{input}))\in R^{1\times 1}$$

where the output channel of $h_{\theta }$ is 1, and the activation function *Sigmoid* is defined as $\frac{1}{1+e^{-x}}\in \left(0,1\right)$ . Finally, the module outputs the learned soft mask $x^{GA}=\{x_{i}^{GA}\;|i\in \left[1,N\right]\}$ .

The reason for designing a global attention mechanism is quite straightforward. Given that each object class possesses distinct feature patterns that may include subtle points such as guitar strings or airplane wings, it’s possible for these feature patterns to be overlooked during the aggregation process, which extracts numerous features. Hence, there is a need to measure the importance of each group $F\left(x_{i}\right)$ denoted as ${x_{i}}^{GA}$ , and weight the global feature g using a learned soft mask ${x_{i}}^{GA}$ .

Furthermore, the reason for incorporating more crucial geometric information (namely, ${\left|\left|x_{j}-x_{i}\;\right|\right|}_{2}$ ) into the global attention module is to expedite and enhance the learning of the global soft mask ${x_{i}}^{GA}$ . While MLPs can theoretically approximate any nonlinear function, such as high-order information and squared Euclidean distance (2nd order: ${\left|\left|x_{j}-x_{i}\;\right|\right|}_{2}^{2}$ ), the literature suggests that models with high-order convolutional filters $\left(\omega_{1}x+\omega_{2}x2+\omega_{3}x\right)$ can achieve higher classification accuracy in several benchmarks [31]. To address the same issue in the proposed model in this paper, additional crucial geometric information (namely, ${\left|\left|x_{j}-x_{i}\;\right|\right|}^{2}$ ) was also chosen to assist the shared MLP in effectively discovering feature patterns and determining the importance of each input point $x_{i}$ denoted as ${x_{i}}^{GA}$ .

### 3.3. The Structure of Att-AdaptNet

**Figure 5:**
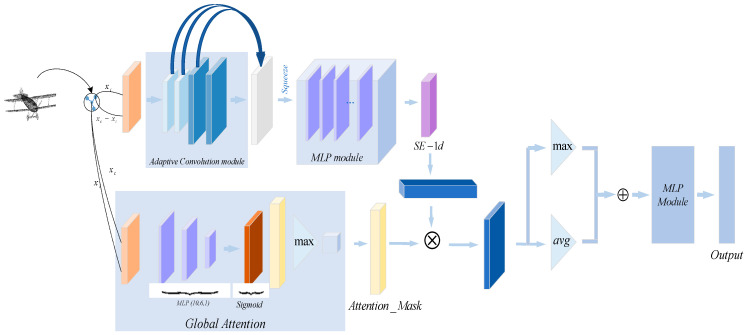
Att-AdaptNet network framework.

After obtaining the mask, denoted as $x^{GA}$ , from the Global Attention Module and the global features, this paper performs element-wise multiplication on them and generates new global features using the ReLU activation function. Following the principles of PointNet for 3D point cloud data classification, most models use max-pooling instead of average-pooling layers. Intuitively, max-pooling should be superior to avg-pooling, as the strongest activation might represent the most prominent feature of a class. However, the results of avg-pooling can also reflect important class features; otherwise, models using average pooling would yield unreasonable results. To gather more valuable information, the experiment chooses to aggregate all points in the global feature regularization using both max-pooling and average-pooling simultaneously. The results of the avg-pooling layer and max-pooling layer are concatenated into a complete classification vector with a dimension of 2048. Finally, a 3-layer MLP is employed to output the classification scores, where C, C/R, and C represent the dimensions of the three neural layers in the MLP, with R being a reduction factor to reduce parameter complexity, as illustrated in Figure 5.

## 4. Experimental Results and Analysis

To assess the effectiveness and robustness of the designed Att-AdaptNet network presented in this paper, a comprehensive set of experiments and corresponding analyses has been conducted in this section. Initially, the proposed Att-AdaptNet network for 3D point cloud classification is primarily validated on the ModelNet40 dataset. It is evaluated by comparing it with other 3D point cloud classification methods on the ModelNet40 benchmark to assess the effectiveness of the approach presented in this chapter. Subsequently, an analysis of the details of the Att-AdaptNet network architecture is performed. Various experiments with different model parameter settings are conducted to determine the optimal parameter configuration that yields the best results.

### 4.1. Datasets

**Figure 6:**
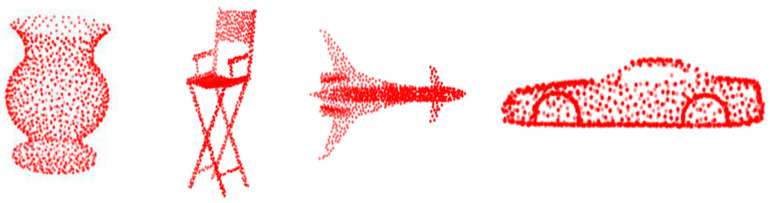
ModelNet40 dataset.

In this study, the Att-AdaptNet is evaluated using the publicly available ModelNet40 3D point cloud dataset. This dataset comprises 12,311 meshed CAD models from 40 different categories, with 9843 models allocated for training and 2468 models designated for testing purposes. A uniform sampling approach is employed to extract 768 points from each object. Only the (x, y, z) coordinates of these sampled points are used as input data. Figure 6 provides illustrative examples from the ModelNet40 dataset.

### 4.2. Experimental Environment and Parameter Configuration

The Att-AdaptNet architecture, as illustrated in Figure 4, dynamically recalculates the graph based on feature similarity at each layer, with a fixed neighborhood size of 20 for all layers. This method incorporates shortcut connections and aggregates multi-scale features using a shared fully connected layer (1024). The global features are obtained using the max-pooling function. Detailed experimental settings are presented in Table 1.

### 4.3. Analysis of Different ‘k’ Values

**Figure 7:**
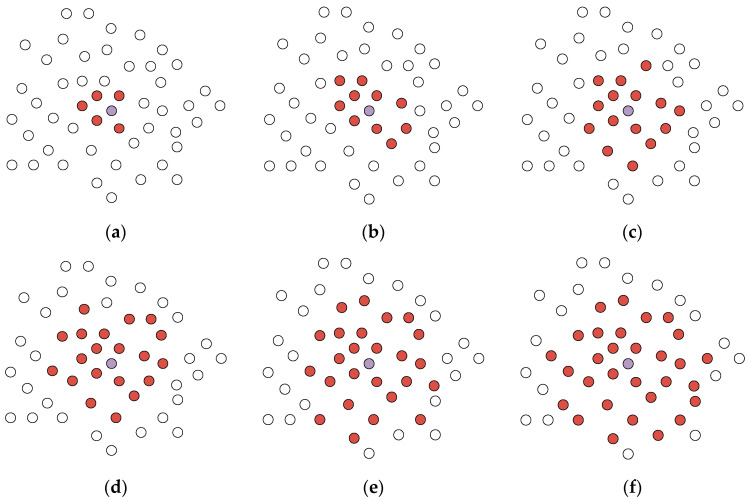
The Distribution of Surrounding Points at Different ‘k’ Values. (**a**) k = 5; (**b**) k = 10; (**c**) k = 15; (**d**) k = 20; (**e**) k = 25; (**f**) k = 30.

In The Adaptive Graph Convolution Module, the neighborhood size (k) is a critical parameter for extracting local geometric features. In this section, we conduct experiments to investigate the influence of different values of ‘k’ on classification accuracy using the ModelNet40 dataset. Table 2 displays the accuracy performance of the model for ‘k’ values of 5, 10, 15, 20, 25, and 30. Figure 7 provides more detail, illustrating the variation in the model’s overall and average accuracy as ‘k’ values range from 5 to 30. In Figure 7, the purple points represent central points, while the red points denote the points surrounding the central points. The attention of central points to their surrounding points is depicted for different values of ‘k’. For example, when k = 5, the central point focuses on the nearest 5 points in its vicinity. Similarly, with k = 10, the central point pays attention to the surrounding 10 points.

**Figure 8:**
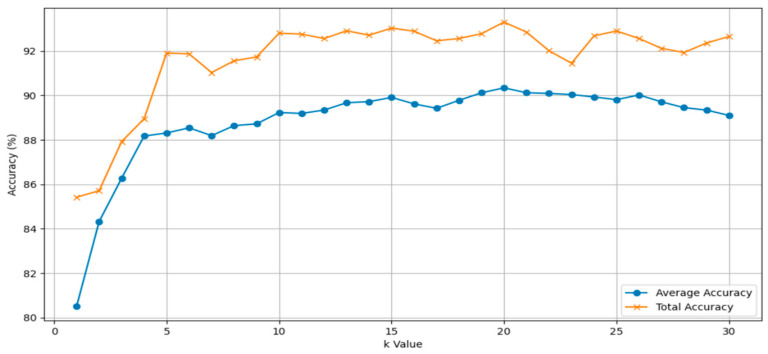
Influence of ‘k’ value on classification accuracy.

As shown in Table 2, the results are notably better when k is set to 20 compared to other values, indicating that the algorithm performs optimally with k = 20. It is worth noting that reducing the number of neighboring points decreases the computational complexity of the algorithm. However, due to the limited receptive field, this reduction negatively impacts the algorithm’s performance. Conversely, larger values of k introduce more noise into the neighborhood. Since local information becomes diluted within larger neighborhoods, it hampers the learning of local geometric features. Consequently, increasing k does not lead to improved performance. Even when k is reduced to 10, the network still achieves relatively good results. But, it can be seen from Figure 8 that when the ‘k’ value is 20, the model effect is optimal, so ‘k’ = 20 is selected as the premise in the following other hyperparameter experiments.

### 4.4. Analysis of Different Point Cloud Numbers

**Figure 9:**
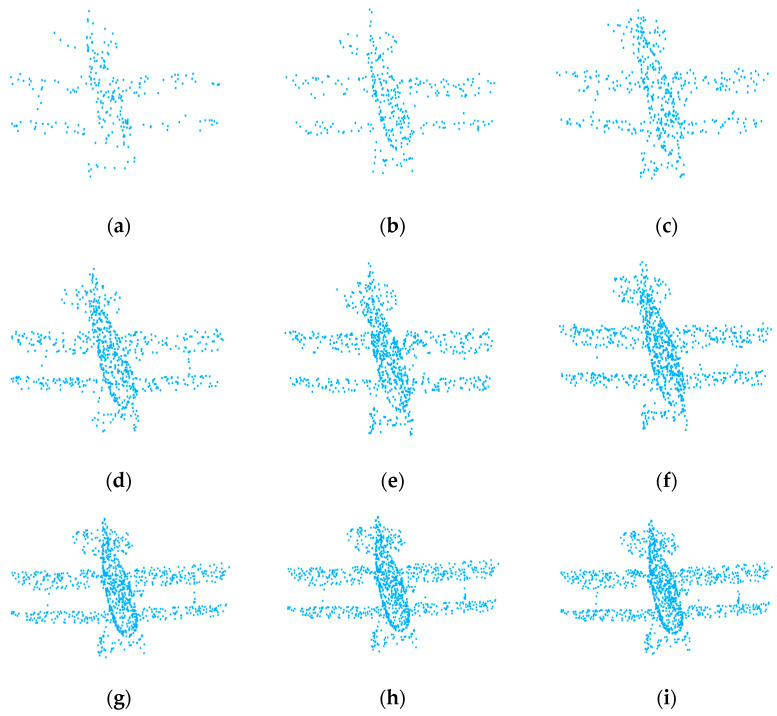
Visualization of different points. (**a**) num_point = 256; (**b**) num_point = 384; (**c**) num_point = 512; (**d**) num_point = 768; (**e**) num_point = 896; (**f**) num_point = 1024; (**g**) num_point = 1152; (**h**) num_point = 1280; (**i**) num_point = 1408.

**Figure 10:**
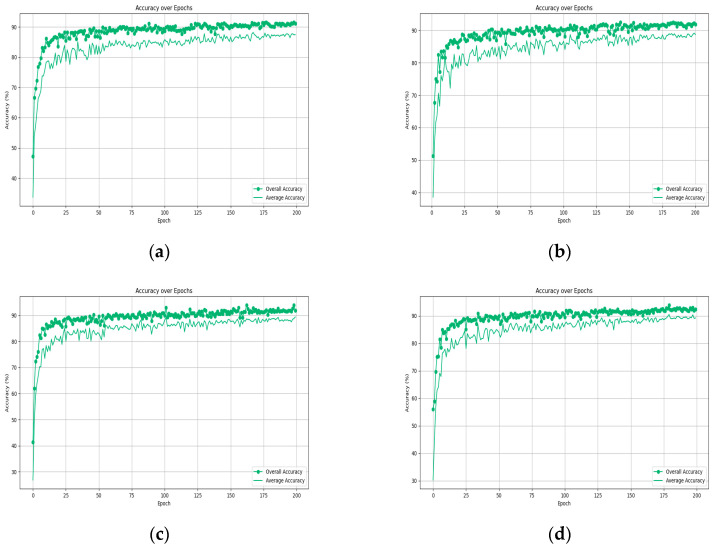

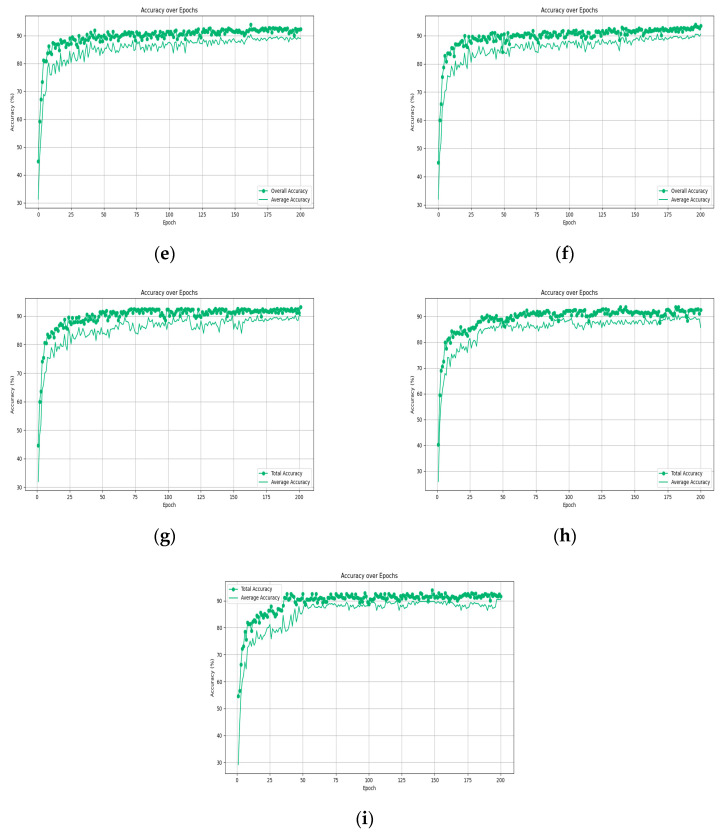
Comparison of classification results of different points. (**a**) Num_point = 256; (**b**) Num_point = 384; (**c**) Num_point = 512; (**d**) Num_point = 768; (**e**) Num_point = 896; (**f**) Num_point = 1024; (**g**) Num_point = 1152; (**h**) Num_point = 1280; (**i**) Num_point = 1408.

The performance of deep learning models is often correlated with the number of features in the used data, generally exhibiting a positive relationship. However, the challenge lies in the fact that for a given dataset and model, this relationship tends to display a trend of initially increasing and then slightly decreasing as the number of individual data features grows. Therefore, identifying the optimal point for this data size is crucial to fully unleash the model’s performance. This paper assesses the robustness of the Att-AdaptNet model on the ModelNet40 dataset. Sparse point clouds with 256, 384, 512, 768, 896, 1024, 1152, 1280, and 1408 points are employed as input to explore the optimal feature count that can fully unleash the model’s performance. During testing, the neighborhood size for all networks is fixed at k = 20. Figure 9 shows an image of the number of clouds at different points. The results of these experiments are presented in Figure 10.

Figure 10 illustrates the significant robustness of the Att-AdaptNet across different point cloud densities, demonstrating its strong resilience. Notably, even with a point count as low as 256, its classification performance surpasses that of PointNet in terms of robustness, achieving an overall accuracy of 91.53% and an average accuracy of 88.17%.

Analysis reveals that as the number of points increases, there is a corresponding rise in both the overall and average accuracy rates of the Att-AdaptNet. With 256 points in the cloud, the model’s overall accuracy hovers around 90%. When the point cloud numbers reach 384, 512, and 768, the overall accuracy consistently exceeds 90% in the middle to later stages of iteration, peaking at 93.57%. Notably, when the model processes 1024 points, its performance is fully realized, achieving the highest overall and average accuracy rates of 93.81% and 90.80%, respectively. However, when the number of point clouds used for training exceeds 1024, specifically at 1152, 1280, and 1408 points, there is a slight decline in the model’s performance. Specifically, when the point cloud counts are 1152, 1280, and 1408, the accuracies are 93.62%, 93.60%, and 93.58%, respectively. In other words, Att-AdaptNet reaches performance saturation at an input feature count of 1024, and an excessive number of features can introduce noise interference to the model. The occurrence of this phenomenon is attributed to the existence of a performance saturation point in the deep learning model. When the number of features becomes excessive, the model may encounter the issue of overfitting, where it overly adapts to the training data, consequently losing its ability to generalize. Additionally, augmenting the number of features can lead to an increase in the computational complexity of the model, prolonging training time, and potentially necessitating more data to mitigate overfitting.

**Figure 11:**
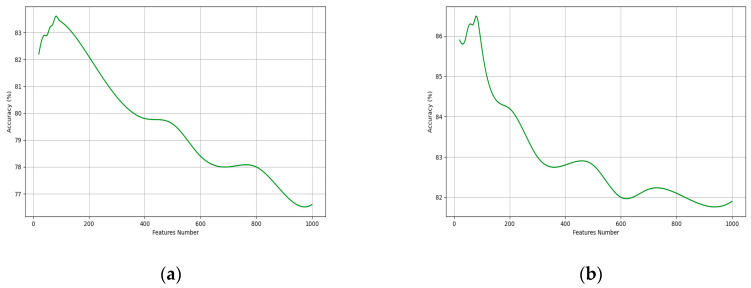
The Impact of Different Feature Number on Model Performance. (**a**) Single-Layer Dense; (**b**) Double-Layer Dense.

Here, we construct a straightforward model to conduct an empirical validation experiment on the existence of performance saturation points. We opt for a single-layer dense and double-layer dense as the model and randomly generate 10,000 data points with lengths ranging from 20 to 1000. The experimental results are illustrated in the following Figure 11.

### 4.5. The Impact of Perceptron Layer Depth on Model Performance

**Figure 12:**
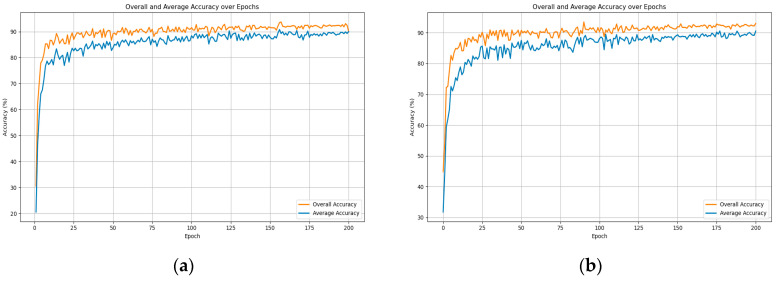

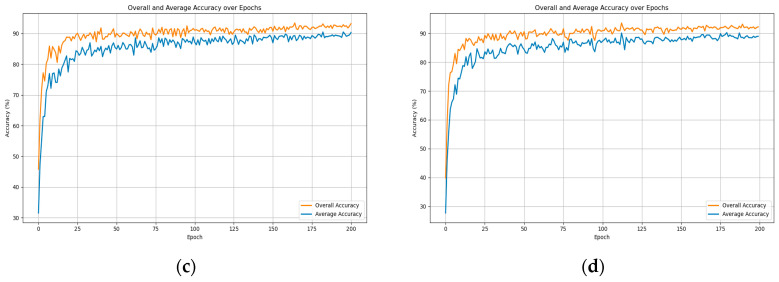
The Impact of Varying Perceptron Layer Depths on the Results. (**a**) layer_number = 3; (**b**) layer_number = 4; (**c**) layer_number = 5; (**d**) layer_number = 6.

In the global attention module, attention masks are generated via a multilayer perceptron in conjunction with normalization layers. The global capacity of the model is, to some extent, contingent upon the number of layers in the perceptron, indicating that the model’s fitting ability is influenced by the depth of the perceptron layers. Thus, this paper has selected 3, 4, 5, and 6 as the layer counts for the perceptron to determine the optimal layer configuration for model performance. The experimental outcomes are presented in Figure 12.

**Figure 13:**
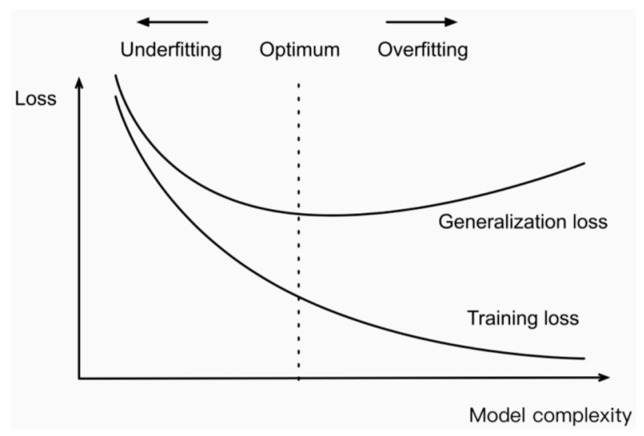
The Relationship Between Model Complexity and Performance.

As shown in Figure 12, when the number of MLP layers is three, the overall accuracy reaches 93.81%, and the average accuracy is 90.80%. However, as the number of layers increases, there is a gradual decrease in overall accuracy, dropping to 93.16% with 6 layers. On the other hand, the average accuracy remains relatively stable within a certain range, albeit with a slight downward trend. This phenomenon can be attributed to the increased complexity of the model structure due to the addition of MLP layers, leading to potential underfitting during training. This prevents the model from fully learning the data distribution patterns and thus limits its performance. The following Figure 13 effectively illustrates the relationship between model complexity and performance. When deep learning models become excessively complex, implying a larger number of training parameters and deeper gradient backpropagation, they may experience underfitting, thereby failing to generalize well to new data and losing their ability to generalize.

### 4.6. Effectiveness of the Proposed Algorithm in 3D Point Cloud Classification

To validate the efficacy of the Att-AdaptNet, this chapter has chosen to compare it with other representative point cloud classification models under identical experimental conditions using the ModelNet40 dataset. The evaluation is primarily based on overall classification accuracy and average classification accuracy, with the precision of classifying 3D point cloud shapes as the evaluation criterion. Brief information on each model is presented as follows.

(1) PointNet: It is comprised of Multi-Layer Perceptrons (MLPs), maxpooling layers, and fully connected layers, capable of directly processing point clouds and extracting spatial features for classification tasks.
(2) PointNet++: It is an advanced model that builds upon the original PointNet architecture, introducing hierarchical neural networks and utilizing a set abstraction layer to capture local structures at multiple scales, enabling more effective processing of spatially distributed data in point clouds.
(3) PCNN: The framework consists of two operators: extension and restriction, mapping point cloud functions to volumetric functions and vise versa. A point cloud convolution is defined by pull-back of the Euclidean volumetric convolution via an extension-restriction mechanism.
(4) GGM-Net: The central component of GGM-Net revolves around extracting features through geometric moments, a process known as GGM convolution. This method involves learning point-specific features and local characteristics from the first- and second-order geometric moments of a point and its immediate neighbors. These learned features are then integrated using an additive approach.
(5) GAPNet: Local geometric representations are learned by embedding a graph attention mechanism within stacked MLPs layers.
(6) FatNet: Presents a new neural network layer, known as the FAT layer, designed to integrate both global point-based and local edge-based features, thereby producing more effective embedding representations.
(7) CT-BLOCK: In the CT-block, two distinct branches are integrated: the ‘C’ branch, signifying the convolution aspect, and the ‘T’ branch, representing the transformer aspect. The convolution branch focuses on executing convolutions on gathered neighboring points to derive local features. Concurrently, the transformer branch applies an offset-attention mechanism to the entire point cloud, facilitating the extraction of global features.
(8) DI-PointCNN: The feature extractor obtains high-dimensional features, while the feature comparator aggregates and disperses homogenous and heterogeneous point clouds in the feature space, respectively. The feature analyzer then completes the task.
(9) DGCNN: A novel neural network module named EdgeConv is proposed, which incorporates local neighborhood information and can be stacked to learn global shape attributes. In a multi-layered system, the affinities in the feature space capture semantic features that may span long distances in the original embeddings.
(10) AGCNN: A graph-based neural network with an attention pooling strategy, termed AGNet, is proposed, capable of extracting local feature information through the construction of topological structures.
(11) Point-Transformer: The Point Transformer model introduces dot-product and point convolution operations, overcoming the limitations of traditional 3D CNNs in processing point cloud data, and offers enhanced flexibility and scalability.
(12) UFO-Net: An efficient local feature learning module is employed as a bridging technique to connect diverse feature extraction modules. UFO-Net utilizes multiple stacked blocks to better capture the feature representations of point clouds.
(13) APES: An attention-based, non-generative point cloud edge sampling method (APES), inspired by the image Canny edge detection algorithm and aided by attention mechanisms.
(14) ULIP + PointNet++: ULIP employs a pre-trained visual-language model, which has already learned a common visual and textual space through extensive training on a vast number of image-text pairs. Subsequently, ULIP utilizes a small set of automatically synthesized triplets to learn a 3D representation space aligned with the public image-text space.

Table 3 presents a comparison of our model with other state-of-the-art (SOTA) models. It is evident that with the widespread application of deep learning in point cloud tasks, the performance in point cloud classification has improved significantly over time. Initially, point cloud data was processed using multi-layer perceptrons, but in recent years, different sampling methods have been utilized. PointNet and PointNet++ marked the beginning, achieving overall accuracies of 89.2% and 91.9% respectively. However, subsequent models like PCNN, GGM-Net, GAPNet, and FATNet have achieved even more advanced results. Recent models such as UFO-Net and APES have reached overall accuracies of 93.5% and above. Att-AdaptNet also demonstrates excellent performance, with an overall accuracy of 93.8% and an average accuracy of 90.8%.

### 4.7. The Effects of Various Attention Mechanisms

To validate the enhancement of model performance by the global attention mechanism proposed in this paper, this chapter selects self-attention and multi-head attention, as reference objects. Additionally, a version of the model without global attention is also set up for comparative experimentation. The experimental results are presented in the following Table 4.

Table 4 reveals that the Self-Attention has a subtle impact on AdaptNet, but Multihead-Attention, conversely, has an adverse effect. This is attributed to the fact that in the Multihead-Attention module, the number of parameters increases multiplicatively with the number of heads, which is not favorable for experiments without massive data volumes. This can lead to insufficient learning in the model, preventing it from fully realizing its potential. In contrast, the global attention mechanism, with its simple linear structure and fewer parameters, demonstrates its advantages. It effectively learns and complements AdaptNet, thus achieving commendable performance on ModelNet40 dataset.

### 4.8. Ablation Experiments

To validate the superiority of the Att-AdaptNet model, an ablation experiment was conducted. Given the relatively simple modular structure of this model, three sets of experiments were chosen for ablation research, namely Adapt, Adapt-MLP, and Att-Adapt. The research results are presented in Table 5, clearly demonstrating that performance of Att-AdaptNet significantly outperforms the other two.

## 5. Conclusions

This paper presents a three-dimensional point cloud shape classification method based on adaptive graph convolution and attention mechanisms. Considering the limitations of existing models in capturing feature information from specific point cloud positions and the inherent constraints of feature learning, a combination of adaptive graph convolution and global attention mechanism is proposed. This approach allows for a more focused exploration of valuable information while effectively addressing issues related to feature loss and detail information preservation, ultimately enhancing classification accuracy. Multiple experiments were conducted on the ModelNet40 dataset to determine the optimal experimental parameter settings for achieving the highest classification accuracy. Compared to other methods, the framework proposed in this paper demonstrates superior classification accuracy, although it comes with longer training times. Future research will explore methods to reduce the training time while maintaining the model’s performance.

## Figures and Tables

**Table 1:** Experiment parameter settings.

Experimental Environment		Model Parameter	
Environment	Configuration	Parameter	Configuration
CPU	Ryzen 5 2400G	Batch size	16
GPU	NVIDIA RTX A2000	Point cloud	1024
RAM	12G	Max epoch	200
Operating system	Window10	Optimizer	SGD
Programming Language	Python3.8	Learning rate	0.001
Deep Learning Framework	Pytorch1.1	Momentum	0.9

**Table 2:** Influence of ‘k’ values on classification accuracy (%).

‘k’ Value	Average Accuracy	Total Accuracy
5	88.31	91.91
10	89.23	92.80
15	89.91	93.03
20	90.34	93.32
25	89.81	92.90
30	89.10	92.65

**Table 3:** Comparison to state-of-the-art model on the ModelNet40 dataset (%).

Model	Accuracy	
	Average Accuracy	Overall Accuracy
PointNet [1]	86.2	89.2
PointNet++ [2]	-	91.9
PCNN [4]	88.1	92.2
GGM-Net [27]	89.0	92.6
GAPNet [25]	89.7	92.4
FatNet [32]	90.6	93.2
CT-BLOCK [33]	90.8	93.5
DI-PointCNN [34]	88.3	92.1
DGCNN [5]	90.2	92.9
AGCNN [35]	90.7	93.4
Point-Transformer [11]	90.6	93.7
UFO-Net [36]	90.8	93.7
APES [37]	-	93.5
ULIP + PointNet++ [38]	-	93.4
**Att-AdaptNet (Ours)**	**90.8**	**93.8**

**Table 4:** The Impact of Different Attention Mechanisms on Model Performance.

Model	Average Accuracy	Total Accuracy
AdaptNet	90.7	93.4
AdaptNet + Self-Attention	90.7	93.5
AdaptNet + Multihead Attention	90.5	93.4
**Att-AdaptNet (Ours)**	**90.8**	**93.8**

**Table 5:** Ablation Experiments Result.

Model	Average Accuracy	Total Accuracy
Adapt	90.5	93.2
Adapt-MLP	90.7	93.4
**Att-AdaptNet (Ours)**	**90.8**	**93.8**

## Data Availability

This study is an experimental analysis of a publicly dataset.
